# The INGV data registry as a curated metadata infrastructure for Earth Science data stewardship

**DOI:** 10.1038/s41597-026-06980-3

**Published:** 2026-03-05

**Authors:** Mario Locati, Salvatore Mazza, Placido Montalto, Vincenzo Romano, Valentino Lauciani, Roberto Vallone, Stefano Cacciaguerra

**Affiliations:** 1 Istituto Nazionale di Geofisica e Vulcanologia (INGV), Sezione di Milano, Milan, 20133 Italy; 2 Istituto Nazionale di Geofisica e Vulcanologia (INGV), Osservatorio Nazionale Terremoti, Rome, 00143 Italy; 3 Istituto Nazionale di Geofisica e Vulcanologia (INGV), Osservatorio Etneo, Catania, 95125 Italy; 4 Istituto Nazionale di Geofisica e Vulcanologia (INGV), Sezione di Roma 2, Rome, 00143 Italy; 5 Istituto Nazionale di Geofisica e Vulcanologia (INGV), Sezione di Roma 1, Rome, 00143 Italy; 6 Istituto Nazionale di Geofisica e Vulcanologia (INGV), Sezione di Bologna, Bologna, 40127 Italy

**Keywords:** Solid Earth sciences, Geophysics

## Abstract

The Istituto Nazionale di Geofisica e Vulcanologia (INGV), Italy’s primary institution for geophysics and volcanology, produces vast, heterogeneous geophysical and volcanological datasets. To enhance these assets under Open Science mandates, we implemented the INGV Data Registry: a centralized, curated metadata infrastructure. This metadata-only system is designed to decouple data description from physical storage, providing a scalable solution for distributed data environments. The Registry operates as a dynamic ecosystem that manages hundreds of records, each assigned to a Digital Object Identifier (DOI) and mapped to international standards. This article examines the implementation and impact of the Registry as a critical infrastructure that supports the FAIR data principles. We demonstrate how a three-tiered validation workflow, combined with the integration of Persistent Identifiers (ORCIDs, RORs), ensures high-quality, interoperable metadata. By providing a single discovery point for INGV’s distributed data assets, the Registry offers a model for data stewardship in large research organizations, accelerating scientific discovery and decoupling data availability from traditional publication cycles. The underlying metadata dataset is publicly available and formally citable.

## Introduction

The global scientific community’s shift towards Open Science, promoted by initiatives like the European Open Science Cloud (EOSC)^1^, has underscored the need for robust research data management. In alignment with European regulations such as the INSPIRE Directive^2^ and the Open Data Directive^3^, Italy’s Istituto Nazionale di Geofisica e Vulcanologia (INGV) developed a comprehensive data policy^4^. This strategic direction is reinforced by the institute’s commitment to reforming research assessment through CoARA^5^, contributing to the Italian National Plan for Open Science (PNSA)^6^, and adhering to the FAIR Principles^7^.

A primary challenge for large, multi-site organizations such as INGV, which employs nearly 1000 personnel distributed across dozens of offices throughout Italy, is the effective stewardship of diverse research outputs. This challenge is intensified by the institutional requirement to comprehensively map all published data, a task that remains significantly difficult for the central administration due to the high degree of organizational complexity and the geographical fragmentation of its data holdings.

**Fig. 1:**
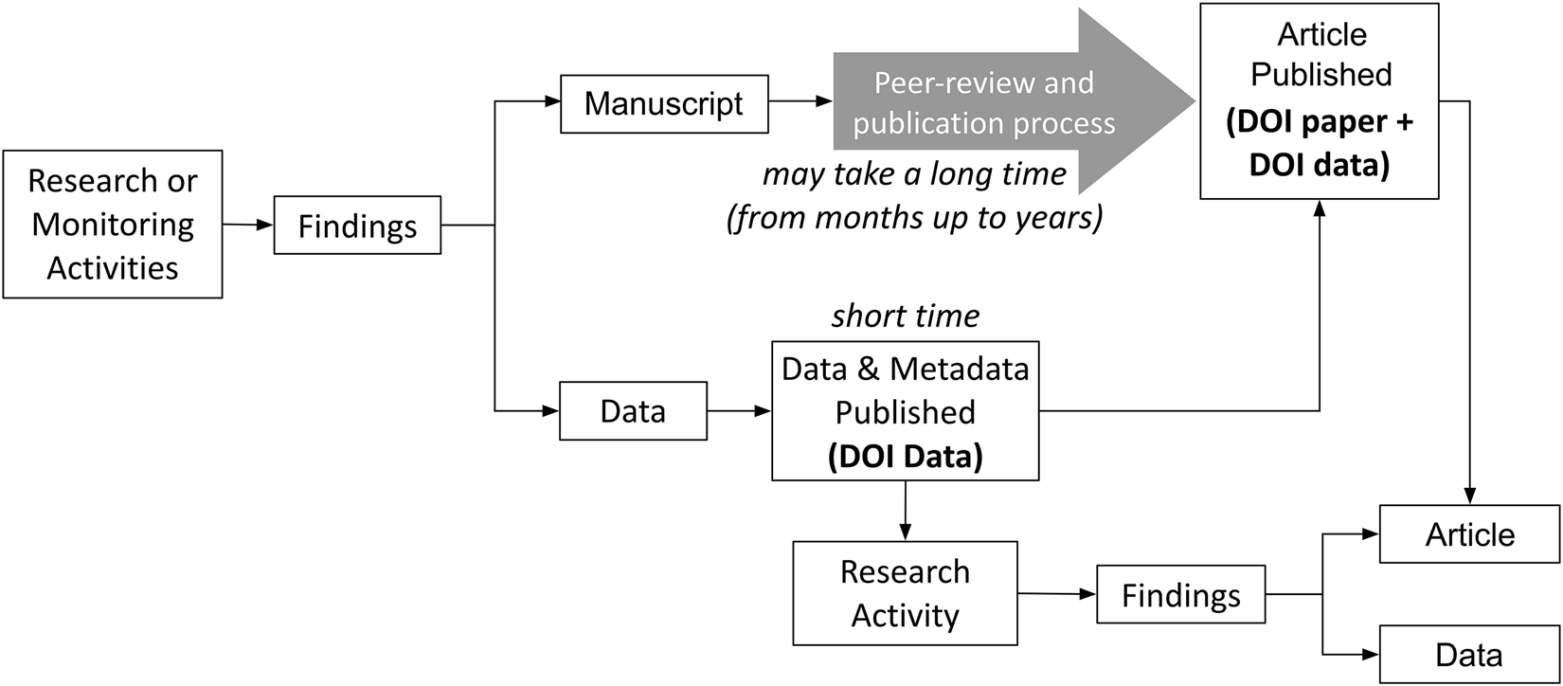
The “data-first” publication principle of the INGV Data Policy. The diagram contrasts two workflows that originate from research activities. The top path represents the traditional scientific article-centric process, in which data are often published alongside the final article after a lengthy peer review. The bottom path illustrates the INGV approach, in which data and metadata are rapidly published with a dedicated DOI. This makes them immediately available for new research activities, well before the associated scientific article is completed.

Traditional workflows centered on scientific articles often delay the availability of data. To address this, INGV adopted a “data-first” (Fig. 1) publication principle, prioritizing the rapid release of data and metadata. The cornerstone of this strategy is the INGV Data Registry^8^, launched in 2019 under the supervision of the institution’s Data Management Office (DMO). Rather than a simple web portal or a data repository, the Registry^8^ represents a curated metadata infrastructure designed to address the challenges of data discovery and navigation in the Earth sciences by providing a centralized index of scientific assets hosted across different platforms.

**Fig. 2:**
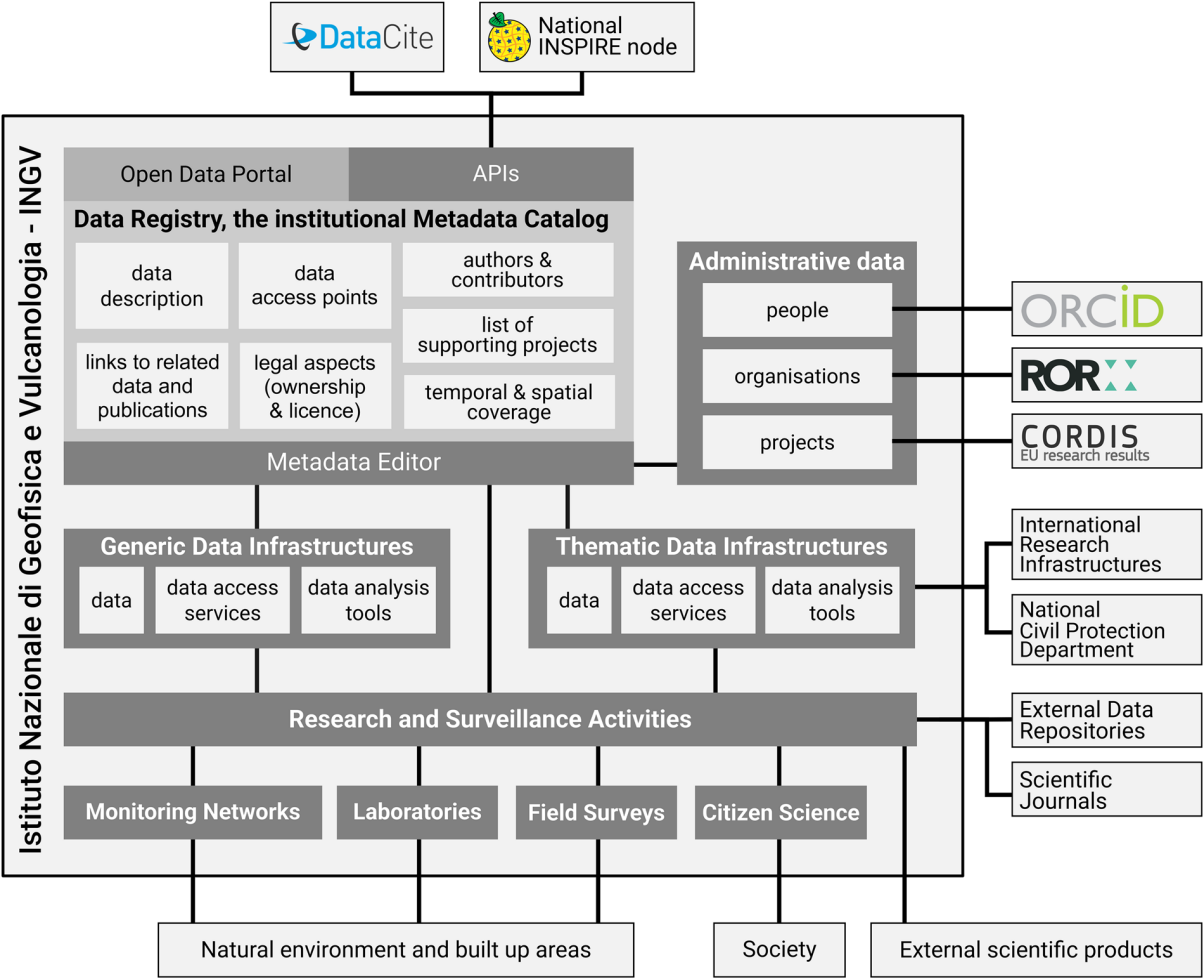
The INGV data ecosystem and the role of the Data Registry. The diagram illustrates the flow of information within the institute, from research activities to the metadata editor and final publication in the Registry and external nodes.

The metadata dataset is designed to maximize interoperability and discoverability of metadata, and it is also tightly integrated into the INGV data ecosystem (Fig. 2). Each record is enriched with multiple metadata standards, including DataCite for citation, ISO 19115/19139 for geospatial information, and DCAT-AP for compatibility with infrastructures like the European Plate Observing System (EPOS)^9–11^.

## Results

### Registry growth and scientific coverage

**Fig. 3:**
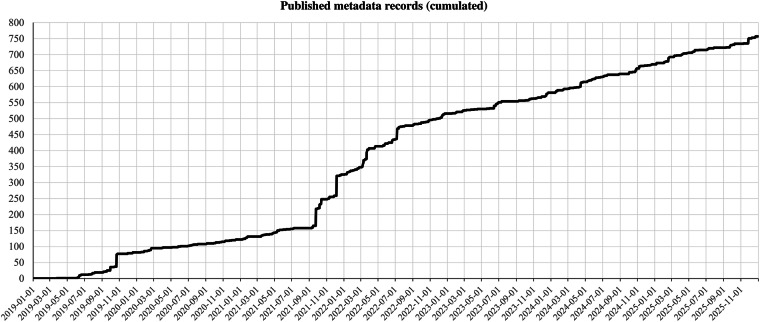
Cumulative growth of published metadata records. The chart shows the total number of validated metadata records in the INGV Data Registry from its launch in January 2019 to December 2025. The steady increase demonstrates the continuous adoption and effectiveness of the validation workflow.

Since its launch in January 2019, the INGV Data Registry^8^ has shown continuous and steady growth in the number of validated metadata records (Fig. 3). As of December 2025, the collection comprises 794 records, representing a significant portion of the institute’s observational and research output.

The distribution of datasets reflects the institute’s multidisciplinary nature. The “Earthquakes” department accounts for 43.5% of the total datasets, followed by the “Environment” (34.6%) and the “Volcanoes” (13.9%) Departments (Table 1). A more in-depth thematic analysis (Table 2) reveals a strong focus on seismological and infrasonic data (45.8%), though significant contributions also come from the geochemical, geophysical, and geological domains. The thematic classes offer a detailed breakdown of the categories available on the public portal homepage (https://data.ingv.it/). Data can also be accessed via subdivisions based on scientific departments (“Earthquakes,” “Environment,” and “Volcanoes”) as primary filters, which better align with the organizational structure. Users can search for specific parameters (e.g., authors, title, description, or DOI) using free-text queries.

**Table 1:** Distribution of the datasets by the INGV scientific department (data updated to December 2025).

INGV Scientific Department	% of Datasets
Earthquakes	43.5
Environment	34.6
Volcanoes	13.9
Earthquakes, Volcanoes	4.0
Volcanoes, Environment	1.8
Earthquakes, Volcanoes, Environment	1.5
Earthquakes, Environment	0.8

**Table 2:** Distribution of the datasets by the thematic classes (data updated to December 2025).

Thematic Class	% of Datasets
Seismological and infrasonic data	44.0
Geochemical data	13.2
Geophysical data	12.2
Geological data	9.9
Data of physical samples	5.4
Geodetic data	4.3
Remote sensing data	2.5
Volcanology data	2.5
Data of atmospheric geophysics and aeronomy	2.3
Data from numerical modeling	2.0

### Operational efficiency and quality control

The implementation of a structured validation workflow has yielded measurable results in terms of metadata quality and system efficiency. It is vital to clarify that DMO validation focuses exclusively on the quality, completeness, and accuracy of the metadata records, while the responsibility for the integrity of the data files remains with the respective hosting repositories. The automated pre-submission checks significantly reduce formal errors (e.g., date formats, ORCID presence). The multi-level human-led validation process - involving formal, scientific, and strategic reviews - ensures that every published metadata record is not only formally correct but also scientifically accountable.

A quantitative analysis of the timestamps in the Registry’s^8^ internal database, which covers all transactions from 2019 to 2025, shows that new records are processed in an average of 11 days, while updates to existing records take an average of 5 days. These metrics, derived from automated tracking of each validation step, suggest an effective balance between rigorous quality control and timely data availability.

The frequency with which researchers send validation requests varies significantly based on environmental and institutional factors. Sudden spikes often occur at the conclusion of major research projects or immediately following natural events, such as seismic sequences or volcanic eruptions. During such emergencies, multiple research groups typically conduct rapid field surveys or deploy additional temporary monitoring networks, resulting in an increase in metadata submissions. Conversely, request volume tends to decrease during extended holiday breaks. Approval timelines may also be influenced by contingent conditions, including administrative reporting deadlines and major international conferences involving the assigned expert validators.

### Interoperability and metadata ecosystem integration

**Fig. 4:**
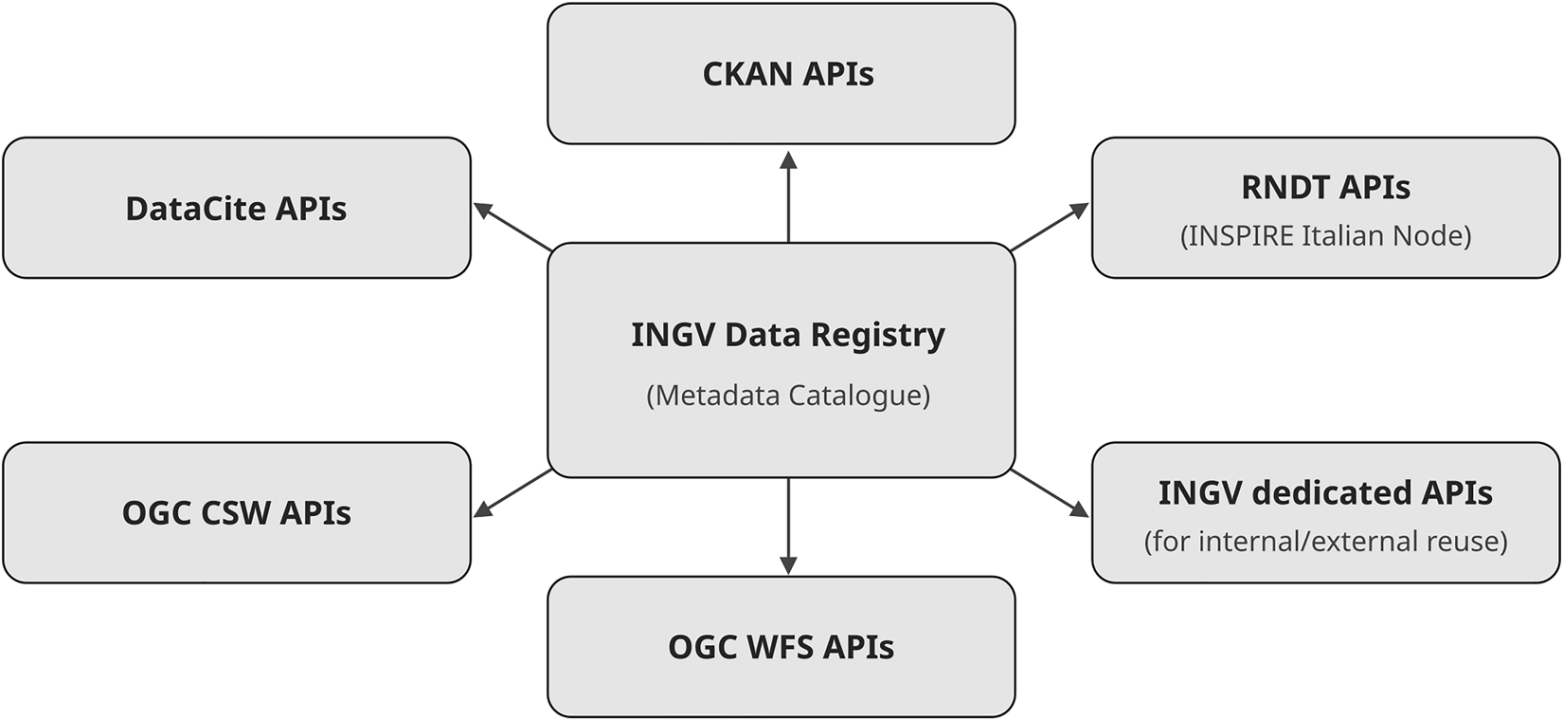
Programmatic access channels to the metadata catalogue. The diagram shows the main APIs through which the metadata contained in the INGV Data Registry can be accessed. These channels are designed to ensure interoperability with a wide range of external platforms, including DataCite, geospatial clients, and national/European portals.

The Registry^8^ serves as a primary hub for external data infrastructures. The system ensures maximum interoperability by exposing multiple metadata standards via dedicated programmatic channels (Fig. 4). Since all records are assigned to a DataCite DOI, they can be discovered and queried via the global DataCite infrastructure APIs and the DataCite Commons platform^12^. This ensures maximum visibility within the global graph of scientific knowledge.

Currently, the Registry^8^ successfully feeds metadata into a variety of scientific infrastructures. These include major European Research Infrastructures (RIs) such as EPOS ERIC^9–11^ and EMSO ERIC^13,14^, as well as domain-specific systems like the Italian node of the European Integrated Data Archive (EIDA)^15^ for seismological waveforms, the TSDSystem framework^16^ for volcanological time series, and the Multidisciplinary Oceanic Information SysTem (MOIST, http://www.moist.it/) or the institutional ERDDAP installation (https://oceano.bo.ingv.it/erddap/) for marine observations. It also provides metadata to generalist infrastructures like EOSC (European Open Science Cloud, https://eosc.eu/), the official European data portal (https://data.europa.eu/), and national catalogs including RNDT (National Catalog for Spatial Data, https://geodati.gov.it/) and the national Open Data Portal (https://dati.gov.it/).

The adoption of PIDs (Persistent Identifiers) has been a key result: each record uniquely links contributors to ORCIDs (Open Researcher and Contributor ID, https://orcid.org/) and involved institutions to ROR (Research Organization Registry, https://ror.org/) IDs, ensuring that INGV’s research contributions are correctly attributed and traceable.

## Discussion

### Impact on data stewardship culture

INGV’s transition to a “data-first” approach has fostered a shift in its internal culture. By treating metadata as citable scientific products, the Registry^8^ formally recognizes researchers’ data management efforts. This acknowledgment operates within the legal framework of the INGV Data Policy^4^, which states that academic credit is given to individual authors -legally defined as “Data Producers”- while the institution retains legal ownership of the research data as the employer of the personnel who produced it. This approach aims to equate the valuation of data publications with that of traditional scientific articles. It formally recognizes the intellectual effort involved in data stewardship, aligning with the principles of CoARA and national Open Science mandates. We observed that promoting these practices is particularly effective among early-career researchers, who are increasingly receptive to the concept that all research outputs deserve proper sharing and valuation. This internal cultural shift mirrors broader international trends in the geosciences promoted either by EPOS ERIC and by the International Federation of Digital Seismic Networks (FDSN); for instance, recent multi-institutional studies on seismological waveform data demonstrate a clear increase in DOI adoption and citation uptake after a decade of community-wide implementation efforts^17^, reinforcing the institutional value of formal data identification systems.

While transitioning from established publication-centric workflows can be slower for more senior staff, the increasing availability of practical tools - such as those enabling automatic citation generation (e.g., https://citation.doi.org/) - has demonstrated the immediate benefits of high-quality metadata. Furthermore, the accessibility of metadata records on the institutional portal (https://data.ingv.it/) streamlines administrative reporting and allows researchers to reuse metadata for the automated generation of personal or project web pages. This practice, actively encouraged by the DMO, effectively implements the “Enter Once, Reuse Often” principle established in the institutional data policy.

### Addressing the data diaspora and fragmentation

Despite the success of the Registry^8^, the “data diaspora” -the distribution of datasets across various external repositories like Zenodo^18^, Figshare, or PANGAEA (Table 3)- presents ongoing challenges for impact tracking. The Registry^8^ mitigates this problem by acting as a curated “Metadata Overlay”. For externally hosted data, the system does not simply mirror existing metadata but applies an enrichment and synchronization strategy.

**Table 3:** Distribution of the datasets described in the Data Registry by type of hosting repository (data updated to December 2025).

Type of repository	Examples	% of Dataset (approx.)
INGV Institutional Servers	Project websites and web-services, Infrastructures websites and web-services	58.7%
External generalist repositories	Zenodo, Figshare	31.9%
External domain repositories	PANGAEA, EarthChem Library (ECL) by Interdisciplinary Earth Data Alliance	9.4%

If significant errors are detected during validation in the metadata of externally hosted data (e.g., missing authors or incorrect funding), DMO curators request that authors personally rectify the information on the external archive’s management system before updating the Registry^8^. For minor discrepancies, such as missing ORCIDs or RORs, the Metadata Editor performs an automatic remapping using internal INGV databases. This ensures that the institutional Open Data Portal (https://data.ingv.it/) presents an enriched and corrected view while maintaining integrity with the original DOI source metadata.

A persistent challenge remains: despite institutional guidelines, some researchers continue to host datasets on external platforms (e.g., Zenodo) without concurrent registration in the Data Registry^8^. This results in reduced institutional visibility for these research outputs. Such oversights are often driven by the time pressures of the peer-review process, where authors may publish datasets hastily to satisfy reviewer demands, inadvertently treating data stewardship as a secondary priority. Furthermore, as the DMO is staffed by active researchers serving as part-time stewards, the office currently lacks the operational capacity for exhaustive monitoring of individual research activities.

To address these evolving requirements and to strengthen institutional accountability, INGV has recently established a dedicated multidisciplinary working group - comprising scientific monitoring, legal, and research communication expertise - tasked with drafting an updated institutional data policy. This initiative aims to align INGV’s stewardship with the latest national and European Open Science mandates while incorporating the operational experience gained by the DMO through the management of the Data Registry^8^. This strategic shift, combined with the development of automated discovery tools using DataCite’s REST APIs, will enforce stricter control over research outputs in the near future while alleviating the monitoring burden on the DMO.

### Research security and institutional accountability

The evolving global geopolitical landscape has recently led to increased scrutiny regarding the security and integrity of research outputs. The European Council Recommendation of 23 May 2024 on “enhancing research security”^19^ and the corresponding national guidelines issued by the Italian Ministry of University and Research^20^ underscore the necessity for research institutions to implement rigorous oversight of shared assets and international collaborations. We argue that the human-in-the-loop validation workflow adopted by the INGV Data Registry^8^ - specifically the multi-level scientific and strategic approvals - serves as a proactive institutional mechanism for ensuring greater accountability. By requiring formal endorsement from Section and Department Directors before any metadata or data is made publicly discoverable with an institutional DOI, INGV ensures that Open Science goals are consistently balanced with the protection of institutional interests and the mitigation of risks related to research security, thereby operationalizing the foundational principle of being “as open as possible, as closed as necessary”.

This accountability also extends to the protection of personal data under the European General Data Protection Regulation (GDPR)^21^. A notable application is the “Hai Sentito il Terremoto?” (in English “Did you feel the earthquake?”) database^22^, where the validation workflow ensures that questionnaires voluntarily completed by the public are fully anonymized prior to publication, balancing the scientific value of citizen science with legal privacy requirements.

### Challenges and future directions

The future evolution of the Registry^8^ is not merely a technical endeavor but a primary strategic objective for the institution. As previously noted, a dedicated working group has been established to integrate these objectives into a revised Data Policy and an updated implementation framework. This process involves a strategic reconfiguration of the DMO to better align with institutional goals, ensuring the Registry^8^ remains a vital resource for researchers and a high-value asset for the organization.

As the number of metadata records grows in the Registry^8^, addressing information overload is becoming critical. Current feedback highlights that users, especially those external to INGV, often find it challenging to discover relevant assets within the existing flat catalog structure without prior knowledge of specific datasets. To address this, it is necessary to reimagine the Registry’s^8^ front-end architecture, moving toward an intuitive graphical discovery system. This potential evolution would aim to replace the linear listing with interactive thematic collections and visual exploration paths, facilitating deeper integration with emerging institutional repositories like OEdataRep^23,24^ and ensuring that INGV’s data holdings are accessible and discoverable not only to domain specialists but also to the wider research community. Furthermore, as users increasingly rely on Artificial Intelligence tools for information retrieval rather than manual web browsing, it is vital to study and optimize how the Registry^8^ exposes its content. Ensuring that AI algorithms are accurately guided to the correct information without errors is a growing priority for institutional data stewardship. To further ensure metadata quality, we are also experimenting with automated FAIRness evaluation tools, such as the F-UJI tool^25^, which provides real-time feedback on technical completeness and discoverability.

The most significant long-term challenge remains the “last mile” of data sharing: not only simplifying standardized access to actual data through international web services but also concurrently addressing the deeper layers of interoperability. Achieving this requires supporting the scientific community in developing and utilizing shared vocabularies and ontologies directly during the data encoding process. This task is particularly challenging in disciplines still operating within traditional “silos”, where cross-domain semantic alignment is not yet standard practice. While the Registry^8^ already links to existing specialized endpoints facilitating data access via web services at https://data.ingv.it/metadata/web_service_eng, these currently cover only a portion of the institute’s production. Looking forward, INGV aims to bridge these gaps by fostering awareness and providing tools for semantic data management, balancing the costly technical effort with the strategic necessity of making data truly machine-actionable and reusable.

## Methods

### Infrastructure architecture

The INGV Data Registry^8^ infrastructure is built on a modular, containerized architecture using Docker. The central component is a bespoke internal Metadata Editor developed internally at INGV using PHP, JavaScript, and a MySQL database, accessible only to INGV personnel via Google Authentication. This choice was driven by the need to integrate tightly with INGV’s administrative databases for personnel, organizations, and funding projects (Fig. 2). At present, the system operates on a single geographic node, employing regular local backup procedures and RAID 5 disk redundancy to safeguard against hardware failure. To further elevate security and service continuity, a geographic redundancy plan is currently under development to replicate the Registry^8^ across multiple, physically distinct institutional branches of INGV.

The Data Registry^8^ is an integral component of the Institutional Data Policy^4^ established in 2018. Following an initial pilot phase, it has become a defining strategic asset for the organization. Consequently, the infrastructure supporting the Registry^8^ is not tied to temporary projects but is integrated into institutional core activities, with maintenance sustainably guaranteed over the long term through institutional funding. In addition to ensuring infrastructure longevity, INGV is committed to preserving the usability and portability of metadata content over time; the adoption of multiple international standards is specifically intended to facilitate long-term interoperability. Regarding the actual research data described by these metadata records, the current data policy is undergoing revision to design and implement an institutional generalist data repository, leveraging and expanding on successful pilot initiatives such as OEdataRep^23,24^.

### Metadata standards and enrichment

The system implements a multi-standard approach (DataCite, ISO 19115/19139, DCAT-AP). The Registry^8^ manages metadata through a normalized relational schema that acts as a standard-neutral internal representation. Rather than adhering to a single metadata schema, information is logically distributed across dedicated tables for core entities, such as details describing the dataset, authors, research projects, and participating organizations. This architecture allows the system to dynamically reassemble and map these data elements on-the-fly only when a specific output standard is required for dissemination.

This decoupling is essential because metadata standards only partially overlap; for instance, ISO 19115 served via the Open Geospatial Consortium CSW (Catalog Service for the Web) API is significantly more extended than the DataCite metadata schema in some areas. For example, it allows describing multiple technical solutions for machine-actionable data access, particularly regarding services defined by the Open Geospatial Consortium itself such as Web Feature Services (WFS) or Web Map Services (WMS). Conversely, DataCite generally points to a landing page, which limits the ability to provide precise, machine-readable instructions on how the actual data can be accessed. On the other hand, while ISO 19115 provides generic mechanisms for linking related digital objects, the DataCite schema’s primary strength lies in its robust support for “related identifiers”. This feature enables seamless, machine-actionable connections between datasets and other research products, such as data, articles, and software, within the global Persistent Identifier (PID) Graph. The PID Graph is an interconnected network that links various research entities, including researchers, institutions, datasets, and publications, via their persistent identifiers. This enables the identification of intricate relationships and the tracking of extensive research impact^26^. By maintaining a rich internal structure, the Registry^8^ ensures that no granularity is lost, providing the most detailed metadata possible according to the specific capabilities of each target metadata standard.

This architectural choice provides significant long-term extensibility, allowing the infrastructure to adapt to the evolving Open Science landscape without requiring structural database migrations or refactoring. The practical validity of this approach has been demonstrated over the years by the minimal technical overhead required to support successive versions of the DataCite and ISO 19115 standards. Since the Registry’s^8^ inception, these updates have been implemented solely through the adjustment of dynamic mapping layers, ensuring metadata persistence regardless of evolving external standards.

To manage technical complexity, the Metadata Editor organizes the web form for every record into 15 shared thematic groups, regardless of the data’s physical location: (1) Title and Landing Page, (2) Other titles, (3) Data producers, (4) DOI, (5) INGV-specific management details, (6) Details about data, (7) Temporal information, (8) Geographical coverage, (9) Access and distribution, (10) Descriptive texts, (11) Ownership and licenses, (12) Funding projects, (13) Relations to other research products, (14) Relations to research infrastructures, and (15) General notes. This organization is strictly implemented at the Graphical User Interface (GUI) level to enhance usability and does not mirror the underlying normalized database structure. Because these groups are decoupled from the data schema, the layout is highly customizable based on compiler feedback; indeed, the GUI arrangement and field labels have been refined dozens of times over the years without necessitating backend database refactoring.

A fundamental technical distinction concerns how compilers fill up these shared thematic groups, a distinction based on where the actual data resides:

1. Metadata for data in internal repositories. For data hosted on INGV infrastructures, the Metadata Editor allows full control and the application of all DataCite, DCAT-AP, and INSPIRE/ISO 19115 metadata, ensuring high-level native FAIRness. To optimize the data entry process, compilers can initialize a new metadata record through three different paths: (i) creating a blank record from scratch; (ii) cloning an existing record from the Data Registry^8^ to facilitate updates for new dataset versions; or (iii) pre-filling the record by providing a DataCite DOI, which triggers an automated metadata harvest regardless of whether the identifier was previously registered in the Registry^8^. The author manually populates all required fields across the 15 thematic groups.
2. Metadata for data in external repositories. When registering datasets already hosted on external platforms (e.g., Zenodo, Figshare, PANGAEA), the registration process is initiated by providing the DOI assigned by the external hosting organization. The Metadata Editor leverages this identifier to automatically harvest core metadata via the DataCite API. To avoid inconsistencies between the Registry^8^ and the primary DOI source, the fields mapped from DataCite are locked in the web form disabling their editing. However, the Editor allows enrichment by mapping names to internal personnel databases (to associate ORCID and ROR) and by manually completing the remaining thematic groups such as geographical coverage or specific ISO 19115 details that are not provided by the DataCite metadata schema.

The resulting metadata corpus exhibits a degree of heterogeneity in quality. Metadata curated natively within the Registry^8^, often through iterative consultation with the Data Management Office, consistently achieve higher standards of completeness and granularity compared to those harvested from external repositories. In the latter case, unsupervised metadata entry frequently leads to lower-quality records that lack the depth of curated INGV entries.

While the Registry’s^8^ primary objective is to comprehensively map all data published by INGV researchers, achieving uniform high-quality metadata remains an ongoing challenge. Currently, the Data Management Office operates in an advisory capacity, promoting best practices rather than enforcing strict compliance, a limitation that is a central focus of the ongoing revision of the INGV Data Policy. Nevertheless, the institute is investing in long-term data culture through specialized training and seminars, with the goal of fostering a research environment where scientists are increasingly aware of their role in ensuring high-quality metadata as a prerequisite for scientific reproducibility

For technical categorization, we adopt the “Data Processing Levels (DPL)” from the EPOS ERIC Data Policy^27^, classifying data from raw (DPL 0) to integrated products (DPL 3), which closely reflects the level of human-driven intellectual contribution.

To minimize errors from manual entry during record creation, the Metadata Editor retrieves author details from an internal, curated database instead of performing automated name queries on external APIs, which are prone to error. INGV personnel manage their own profiles within this system by securely logging in and manually linking their ORCID IDs and other academic identifiers (e.g., Scopus ID or Researcher ID). For external co-authors, the Data Management Office (DMO) applies a human-in-the-loop approach. DMO curators engage with INGV authors to encourage their external peers to obtain and provide an ORCID ID. ROR identifiers for organizations are mapped using internal institutional databases. The use of standardized international vocabularies is limited and mainly concerns the INSPIRE framework, which is specifically designed for geographic data. Regarding the thematic classes presented in Table 2, they do not strictly follow a specific standard controlled vocabulary at this time. Instead, these classes represent a scientific classification derived from internal surveys of available data conducted during the initial drafting of the INGV Data Policy between 2015 and 2018. However, the aforementioned institutional working group dedicated to updating the institutional data policy is currently evaluating a revision of this classification. Currently, vocabulary adoption is also closely linked to the ongoing development within EPOS ERIC, which, particularly in the fields of seismology and volcanology, remains somewhat slow compared to more advanced disciplines such as geology and marine science.

### Institutional governance and metadata quality assurance workflow

To streamline the curation process and ensure adherence to international standards, the Metadata Editor integrates an automated validation suite. Researchers can execute a comprehensive diagnostic check at any stage of compilation; however, this procedure is a mandatory prerequisite for formal submission. The suite generates a diagnostic report that categorizes issues into two tiers: blocking errors and non-blocking warnings:

- Blocking errors represent critical failures in meeting the baseline requirements for FAIR compliance and institutional policy. These include the omission of ORCIDs for primary authors, missing temporal or geographical coverage, or the lack of ownership and licensing information. A strict enforcement mechanism ensures that the formal validation request to the Data Management Office (DMO) remains disabled until all blocking errors are resolved.
- Non-blocking warnings provide recommendations to enhance metadata richness, typically flagging missing relationships to related research products (e.g., publications, datasets) or the absence of funding project details. While these do not halt the workflow, compilers are encouraged to address them to maximize the discoverability and reusability of the asset.

A distinctive feature of the suite is the remote consistency check performed via an automated crawler. The system verifies that the landing page -the final destination to which the DOI resolves- meets institutional requirements, such as including a backlink to the INGV portal, a ready-to-use bibliographic citation, and explicit licensing terms. Importantly, the automated tool does not distinguish between pages hosted on INGV systems and those on third-party infrastructures where researchers have limited control over layout and content. For this reason, these findings are carefully contextualized during the subsequent manual validation steps. Furthermore, as complex landing page architectures may occasionally cause the crawler to fail or return inaccurate results, this technical limitation is clearly communicated to users to ensure they interpret the diagnostic output as a non-definitive guide.

Overall, this automated scrutiny significantly reduces the administrative burden on the DMO by addressing common errors at the point of entry, ensuring that only high-quality, machine-readable records proceed to manual review.

The Registry^8^ is governed by the Data Management Office (DMO, in Italian “Ufficio Gestione Dati”), established in 2018. This office comprises representatives from each scientific department and technical services who are themselves active researchers. Consequently, they serve in the DMO on a part-time basis, balancing institutional data stewardship with their own research activities. This dual role ensures that the infrastructure evolves in response to both technological trends and the specific needs of the research communities. The DMO operates under a permanent institutional mandate from the INGV presidency, which further secures the long-term maintenance and strategic evolution of the infrastructure.

For each dataset, contact information refers to a scientific and technical curator, ensuring that validators and the DMO have a direct point of contact for any necessary metadata revisions.

The validation process is designed to reflect INGV’s matrix organizational structure, which balances local operational oversight with national scientific strategy. Section Directors lead the institution’s territorial branches, which include offices in cities such as Rome, Milan, Bologna, Pisa, Naples, Palermo and Catania, and serve as the primary figures responsible for the research infrastructures and personnel generating the data. Notably, the INGV Data Policy has established the quantification of datasets published by the personnel affiliated with each section as a key performance indicator for the annual evaluation of Section Directors, thereby institutionalizing data stewardship at the administrative level. In contrast, Department Directors oversee the three national thematic pillars of Earthquakes, Volcanoes, and Environment, focusing on high-level scientific strategy and institutional coordination. This dual oversight ensures that metadata are validated both at the source of production by the infrastructure managers and at the strategic level for national alignment.

**Fig. 5:**
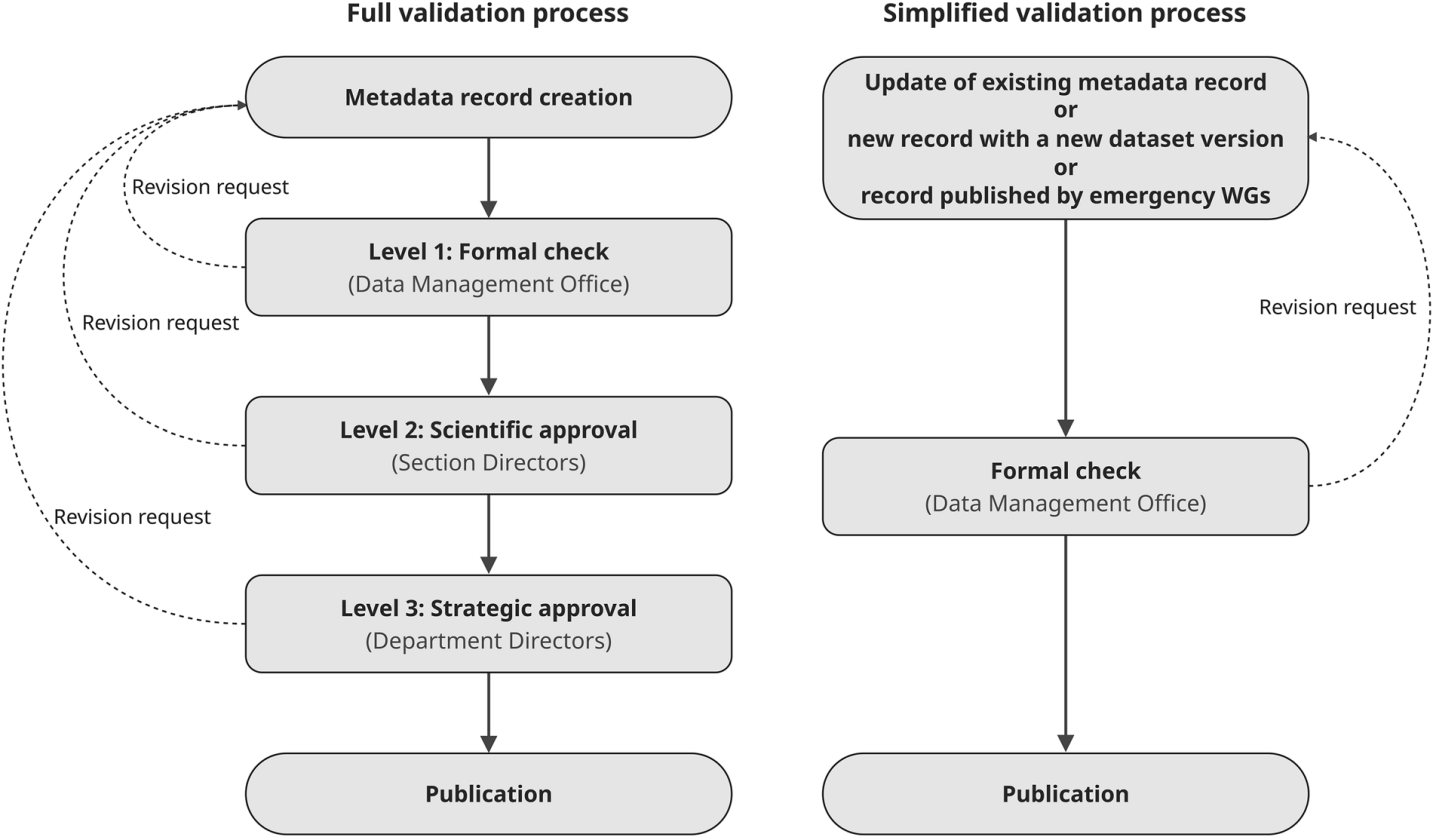
The metadata validation workflow. The flowchart shows the sequential levels of review (Formal, Scientific, Strategic) and the simplified process for updates.

After all the blocking errors in the metadata record have been automatically detected and cleared, the compiler can initiate the validation request. This triggers an automated workflow that manages sequential steps and sends automatic notifications to the relevant personnel. The validation process follows a sequential human-in-the-loop workflow (Fig. 5). Each metadata record is assigned to a scientific and technical curator, who is responsible for its accuracy and may delegate editing permissions to colleagues. The system manages three validation steps:

1. Formal check: Managed by the DMO, this stage verifies metadata completeness and ensures the landing page is accessible, follows policy guidelines, and provides access to the data itself. This phase also involves verifying data ownership, which can be complex when datasets involve authors not affiliated with INGV, a frequent scenario in large-scale collaborative research projects.
2. Scientific Approval: Conducted by Section Directors to confirm scientific accuracy, proper authorship, and alignment with the relevant research domain, leveraging their direct oversight of the producing infrastructure and personnel.
3. Strategic Approval: Provided by Department Directors to ensure institutional alignment and adherence to strategic goals before final publication.

Each validation level can return the record to the authors for revision. To maintain operational agility, a simplified single-level workflow is adopted for: (i) metadata update requests for already published records; (ii) new records describing subsequent versions of existing datasets; and (iii) datasets released by emergency task forces during natural events, such as earthquakes or eruptions. In the latter case, rapid dissemination is prioritized to facilitate immediate community analysis while simultaneously safeguarding the contributors’ scientific credit through formal DOI assignment. This approach reflects a commitment to the ethical principles of data sharing in the geosciences^28^.

### DOI minting and dissemination

Upon validation, the Registry^8^ manages DOI association based on the location where the data is hosted. For datasets hosted on internal INGV infrastructures that lack a persistent identifier, the system interacts with the DataCite API to mint or update an institutional DOI. Conversely, for data already archived in external repositories (e.g., Zenodo, Figshare, or specialized domain archives) that independently assign DOIs, the Registry^8^ simply records and preserves the existing identifier provided by the host platform. This ensures consistency and prevents duplication. Validated records are automatically published to the INGV Open Data Portal (https://data.ingv.it) and made available via multiple public APIs (DataCite, CKAN, and OGC CSW/WFS) in a machine-compatible way.

Although the Registry^8^ efficiently handles complex, dynamic data streams, it’s important to note that most metadata records describe static datasets processed using standard procedures for fixed research products.

To address the specific requirements of dynamic data, INGV assigns a single persistent DOI to continuous data streams (e.g., monitoring flows) as long as they remain typologically homogeneous^29^. Crucially, the institutional strategy identifies the resulting data stream as the primary object for DOI assignment rather than the physical monitoring network. This policy is designed to be inclusive of a wide range of environmental monitoring typologies, encompassing networks that integrate various station types - such as geodetic (GNSS), seismic, geochemical, electromagnetic, ionospheric, and environmental sensors - regardless of their internal complexity. Time-segmented DOIs are not assigned to the raw stream; however, if specific time intervals are extracted and reprocessed for specialized analysis, these subsets are registered as new datasets with dedicated DOIs to ensure the reproducibility of derivative works. Derivative products must cite the original source data stream, with these dependencies formally tracked via DataCite’s relational metadata properties.

One notable case involves data from temporary monitoring networks. These datasets are initially registered as dynamic data streams, which allows for early citation and discovery while measurements are ongoing. Once field operations cease and the network is dismantled, however, the dataset formally transitions to a static state because the data flow is no longer actively updated. The ending data stamp metadata is then added. A fundamental governing principle of the Registry^8^ is that this transition is strictly unidirectional; once a dataset is declared static, it cannot return to a dynamic state. This rule is enforced to guarantee the reproducibility of analyses, ensuring that users can rely on the invariability of a static resource rather than a “moving target”, which would otherwise compromise the ability to exactly reproduce scientific results. For data streams from temporary networks, a DOI is assigned proactively as soon as the installation is planned - for instance, immediately after the FDSN (International Federation of Digital Seismograph Networks) network code is granted for seismological deployments^30^. This proactive assignment facilitates data discovery and tracking from the earliest stages of the research lifecycle, a capability that becomes particularly vital during seismic or volcanic emergencies. In such scenarios, multiple research groups often require simultaneous access to live data streams for real-time analysis; early DOI availability ensures that these contributions are formally attributable and reproducible, supporting both scientific research and monitoring activities conducted in coordination with the National Civil Protection authorities^31^.

Within the Registry^8^, the term “versioning” specifically refers to successive revisions of static datasets where a new DOI is issued to rectify errors or integrate data that significantly enhances the previous version. Links between older and newer versions are clearly presented to users in the Open Data Portal, with these relationships formally stored within the “relatedIdentifier” property of the DataCite metadata schema. The INGV Data Policy mandates that once data is published, it must be preserved indefinitely - even if superseded by a newer version - to guarantee scientific reproducibility.

## Data Availability

The primary access point for the metadata dataset described in this article is the INGV Open Data Portal (https://data.ingv.it/), citable via its dedicated persistent identifier: 10.13127/data-registry^8^. Programmatic and specialized access to the Registry’s metadata is provided through several standardized endpoints: • CKAN API (https://data.ingv.it/api/3/) for general discovery and dataset retrieval; • OGC CSW and WFS (https://ogc.ingv.it/) for geospatial metadata consumption; • DataCite RESTful API (https://support.datacite.org/docs/api) and OAI-PMH API (https://support.datacite.org/docs/datacite-oai-pmh) for global infrastructure queries. Metadata for datasets and collections for which INGV has directly minted a DOI can also be browsed and queried directly on the DataCite Commons platform: • Datasets: https://commons.datacite.org/doi.org?query=client.uid:crui.ingv&resource-type=dataset • Collections: https://commons.datacite.org/doi.org?query=client.uid:crui.ingv&resource-type=collection Individual datasets described within the Registry are discoverable through the same portal, each with its own persistent identifiers.

## Code availability

No custom code was used to generate or analyze the metadata dataset described in this article. The bespoke internal Metadata Editor described in the Methods section is an infrastructure management tool tightly integrated with INGV’s internal administrative and personnel databases; therefore, its underlying source code is restricted for institutional security reasons and is not publicly distributed. However, all operational procedures, data schemas, and validation workflows are comprehensively detailed in this article, allowing for their potential reimplementation or adaptation by other organizations. Furthermore, all other tools, standards, and programmatic APIs utilized to build the infrastructure and disseminate the metadata rely on open-source software.
